# Editorial: RNA regulation mechanisms in microbial-host interactions

**DOI:** 10.3389/fcimb.2025.1751814

**Published:** 2025-12-17

**Authors:** Wenxing Li, Sanqi An

**Affiliations:** 1 Department of Systems Biology, Columbia University Irving Medical Center, New York, NY, United States; 2 Life Sciences Institute, Guangxi Medical University, Nanning, China

**Keywords:** RNA, immune activation, microbial, host, cell biology

## Introduction

The interaction between microbes and their hosts represents a dynamic and multifaceted evolutionary arms race, with both parties continually adapting their molecular arsenals. While traditional paradigms focused predominantly on protein-mediated signaling cascades, the past decade has witnessed a fundamental shift in focus, placing RNA biology at the center of this intricate cross-talk. It is now well-established that RNA molecules—encompassing messenger RNAs (mRNAs) and a diverse array of non-coding RNAs (ncRNAs), such as microRNAs (miRNAs), circular RNAs (circRNAs), and long non-coding RNAs (lncRNAs)—serve as critical modulators of cellular responses during infection. Pathogens have evolved sophisticated mechanisms to exploit these molecules and hijack host machinery, while hosts have co-evolved RNA-based surveillance systems to trigger innate and adaptive immunity.

This Research Topic “RNA Regulation Mechanisms in Microbial-Host Interactions” was curated to systematically explore this complex regulatory landscape, highlighting novel molecular mechanisms, technological advancements, and potential therapeutic applications. This Research Topic brought together six high-quality articles that collectively advance our understanding of how RNA regulation shapes the outcomes of microbial infections, spanning bacterial and viral pathogenesis, the development of innovative detection tools, and the emergence of RNA-based therapeutics.

## Epitranscriptomics and RNA profiling in infection

A major frontier in contemporary RNA biology is epitranscriptomics—the study of biochemical modifications on RNA molecules and their functional consequences. In their original research article, Zou et al. employed cutting-edge direct RNA sequencing technology to investigate the macrophage response to Mycobacterium tuberculosis (Mtb) infection. Their comprehensive study revealed multilayered epitranscriptomic remodeling in host cells and identified specific RNA modifications that correlate with distinct infection stages and immune activation states. These findings offer new molecular targets for understanding tuberculosis pathogenesis and may inform the development of host-directed therapies.

Complementing this focus on modified coding RNAs, Miao et al. shifted their attention to the emerging class of non-coding RNAs, specifically circular RNAs (circRNAs), during viral infection. Using Vesicular Stomatitis Virus (VSV) as a model system, they provided comprehensive profiling of both host-derived and virus-encoded circRNAs. Their findings highlight the dynamic differential expression of these exceptionally stable RNA molecules and suggest that they may function as molecular sponges for miRNAs or sequester RNA-binding proteins, thereby fine-tuning the host antiviral response. This study reinforces the emerging concept that circRNAs are functional players in the host-virus interactome rather than mere splicing byproducts or transcriptional noise.

## Endogenous viral elements and host pathology

The interplay between viral sequences and host physiology extends far beyond acute infection episodes. Li et al. presented intriguing findings on the unexpected impact of endogenous viral elements (EVEs) on human health, specifically in the context of glioma pathogenesis. They demonstrated that EVEs—remnants of ancient viral infections integrated into the human genome—can profoundly influence glioma clinical phenotypes by inducing the expression of the stem cell transcription factor OCT4 in host cells. This study elegantly bridges the traditionally separate fields of virology and oncology, suggesting that these “fossilized” viral remnants in our genome are not dormant relics, but rather, they can be reactivated in specific cellular contexts to drive pathological processes. This work provides a novel perspective on the long-term evolutionary and pathological consequences of microbial-host co-evolution, raising important questions about the broader roles of EVEs in human disease.

## Advanced tools for detection and mechanism decoding

Advancing this rapidly evolving field requires robust and innovative methodologies. Two articles in this Research Topic directly addressed the technological needs of the research community. Ghosh et al. provided a comprehensive and timely review of emerging RNA-centric technologies specifically designed to probe RNA-protein interactions in viral systems. Focusing on positive-sense single-stranded RNA (+ssRNA) viruses—which represent major human pathogens—the authors systematically discussed how these cutting-edge tools are essential for decoding viral life cycles and identifying novel antiviral targets. Their review highlights techniques ranging from classic crosslinking and immunoprecipitation methods to state-of-the-art single-molecule imaging approaches.

On the diagnostic and surveillance front, Que et al. introduced the “HPD-Kit,” a comprehensive and user-friendly toolkit for pathogen detection and molecular analysis. In an era where the rapid, accurate, and cost-effective identification of pathogens is critical for public health preparedness and outbreak response, this toolkit represents a practical application of molecular biology principles to provide actionable diagnostic solutions. The HPD-Kit aims to streamline the pathogen detection process and improve real-time surveillance of infectious agents, with particular relevance for resource-limited settings.

## Therapeutic implications and clinical translation

Understanding the fundamental mechanisms of RNA regulation in infection ultimately aims to foster the development of novel therapeutic interventions. He et al. conducted a systematic review and comprehensive bibliometric analysis to examine the global landscape of small interfering RNA (siRNA) therapeutic development. By mapping research trends, identifying knowledge hotspots, and analyzing collaborative networks in this field, the authors illustrated how foundational research into RNA interference mechanisms is progressively being translated into clinical strategies to combat microbial infections. Their analysis confirms that RNA-based therapeutics represent a rapidly maturing field with immense potential to address the growing threat of antimicrobial resistance, offering precision medicine approaches that could complement or even replace traditional antimicrobial agents.

## Future perspectives and research directions

The articles presented in this Research Topic collectively illustrate the remarkable breadth and depth of RNA regulation in microbial-host interactions. From the epitranscriptomic remodeling of immune cells and the circRNA landscapes of viral infections to the pathological roles of endogenous viral elements, these studies reveal that RNA serves as a central architect of the host-pathogen interface.

Looking forward, several exciting avenues warrant further investigation. Future research should increasingly integrate multi-omics approaches, combining epitranscriptomics with proteomics and single-cell genomics to achieve a holistic understanding of host-pathogen dynamics. Advanced spatial transcriptomics and real-time imaging technologies promise to reveal how RNA regulation varies across tissue microenvironments and infection stages. The success of mRNA vaccines against COVID-19 has demonstrated the tremendous potential of RNA-based therapeutics, encouraging the exploration of circRNAs, self-amplifying RNAs, and host-directed therapies that manipulate RNA regulatory networks to enhance immunity while minimizing resistance development. Additionally, artificial intelligence and machine learning approaches will become increasingly important for predicting RNA structures and interactions based on high-throughput data, accelerating the discovery of novel therapeutic targets. Finally, evolutionary perspectives examining how RNA regulatory mechanisms have co-evolved in pathogens and hosts will provide insights into vulnerability points for therapeutic exploitation.

## Conclusion

We hope this Research Topic serves as both a comprehensive resource and a catalyst for future investigations, encouraging researchers worldwide to delve deeper into the “RNA world” and uncover hidden regulatory mechanisms that govern the delicate balance between health and disease.

